# Micro-RNAs, New performers in multiple myeloma bone marrow microenvironment

**DOI:** 10.1186/2050-7771-2-10

**Published:** 2014-05-30

**Authors:** Jahangir Abdi, Lugui Qiu, Hong Chang

**Affiliations:** 1 Division of Molecular and Cellular Biology, Toronto General Research Institute, Toronto, Canada; 2 Department of Laboratory Medicine & Pathobiology, University of Toronto, 172 St George St, Toronto, ON M5R 0A3, Canada; 3 Institute of Hematology and Blood Disease Hospital, Chinese Academy of Medical Sciences and Peking Union Medical College, Tianjin, China; 4 Department of Laboratory Hematology, Laboratory Medicine Program, Toronto General Hospital, University Health Network, 200 Elizabeth Street, 11E-413, Toronto, Ontario M5G 2C4, Canada

**Keywords:** Multiple myeloma, miRNA, Bone marrow stroma, CAMDR

## Abstract

The established interaction between multiple myeloma cells and bone marrow microenvironment components provides malignant cells with various survival, growth and drug resistance signals. As a new concept, identification of miRNAs and their related gene/protein targets, signaling molecules and pathways in the context of bone marrow microenvironment will help understanding more deeply the pathogenesis of the disease and possible mechanisms underlying environment-induced drug resistance. Recent studies suggest that bone marrow stromal cells can modulate some miRNAs (miR-21, miR-15a/16) in multiple myeloma cells through direct adhesion, cytokine secretion or transfer of miRNA-containing exosomes, however; the specific miRNA targets are not clear. In spite of a remarkable progress in understanding myeloma biology and therapy, the disease persists to be hard to treat. This review will discuss the most recent findings on miRNAs expression and function in the context of bone marrow microenvironment highlighting the miRNAs as potential therapeutic targets in multiple myeloma.

## Introduction

To survive and expand, multiple myeloma (MM) tumor cells have to be in a dynamic interaction with their stromal environment [1,2]. The profound effect of bone marrow stroma on malignant cells makes the bulk tumor resistant to drugs and invasive [2,3]. The latter interaction perhaps has been fully investigated in multiple myeloma. However, the not-clearly-determined mechanisms underlying cell adhesion-mediated drug resistance (CAM-DR) in MM cells in the context of bone marrow microenvironment (BMME) components warrants continuous research to allow identification of more potential drug targets. In developing drugs for MM treatment, little focus has been made on disrupting the above interaction, and variety of drug targets have been identified in isolated MM cells in no interaction with BMME [4-6]. Thus these systems cannot provide a more appropriate reflection of MM cells drug response in the bone marrow. miRNAs (miRs) have become an interesting research focus in MM as these molecules are mostly deregulated in MM, can have multiple targets (which may be oncogenes or tumor suppressor genes, e.g. *MYC*, *TP53*, *PTEN*) and have proved to significantly influence MM tumor *in vitro* and *in vivo*[7-12]. However, most findings cannot reflect the miRs biology in BMME context, while results obtained from some related B cells malignancies strongly support the concept that bone marrow stroma would have regulatory effects on some miR-mediated events including CAMDR.

### miRNAs in MM: “small but stunning performers”

miRs belong to a class of small non-coding RNAs (22–25 nt. in length) which repress the expression of their target genes at translational level by binding to 3′UTR through partial sequence complementarity [13]. Recent studies indicate that miRs could play role in MM progression and pathobiology. It has been shown that the expression of some miRs is higher /or lower in MM cells compared to their normal counterparts [5,10,12]. miRs may target various genes involved in controlling cell cycle, proliferation and apoptosis. If the targets are tumor suppressor genes, miRs are considered as oncomiRs contributing to tumor pathogenesis, while some other miRs functioning as tumor suppressors could be downregulated by the oncogenic process. In support of this, Pichiorri et al. demonstrated that miRs 192, 194 and 215 in MM were positive regulators of p53 but repressed in newly diagnosed patients contributing to disease progression [8]. They also demonstrated that MGUS and MM displayed a unique miR expression signature [5]. For instance, miR-32 and miR-17~92 cluster were selectively upregulated in MM cells and HMCLs but not in monoclonal gamopathy of undetermined clinical significance (MGUS) and normal plasma cells. In addition, miRs19-a/b (part of the mir-17~92 cluster) downregulated suppressor of cytokine signaling-1 (SOCS-1) expression (which is normally an inhibitor of IL-6 signaling but frequently silenced in MM) thus maintaining MM cells growth and proliferation. Microarray analysis in another study also revealed a distinct mostly up-regulated miR signature in MM samples and cell lines compared to MGUS and normal plasma cells, with some miRs being associated with specific MM chromosomal isotypes [7]. The latter findings indicate that some miRs may play prognostic roles in MM. More importantly, miRs have also been indicated to mediate drug resistance in MM cells enforcing our understanding of the mechanism of chemoresistance in MM patients [14,15]. In spite of all the findings on miRs in MM with respect to involvement in various functional responses, the mechanism (s) regulating expression and function of miRs in MM has not been explicitly elucidated, but one possible mechanism may be epigenetic control of miR genes [16,17], however; our knowledge of this potential mechanism in MM is still scanty. Nonetheless, studies conducted in recent years on MM miR biology and function, have now opened a new window to MM targeted therapy exploiting miRNA analogues or their antagonists. More interestingly, combination of miRs or their inhibitors enhance anti-myeloma activity of chemotherapeutic drugs [18].

### miR-mediated functions in MM cells in bone marrow microenvironment context

It is now understood that miRs mediate substantial functions in tumor microenvironment (reviewed at [19] (see Table 1). Bone marrow microenvironment (BMME) components especially bone marrow stromal cells and fibronectin protect MM cells against (drug-induced) apoptotic signals. This protective shield occurs mostly due to adhesion of MM cells to BMSCs leading to cytokine and growth factor secretion by later cells (or both), activation of various genes and signaling pathways in both cell types, and induction of cell adhesion mediated drug resistance (CAM-DR) in MM cells [20-22]. Regarding the fact that MM pathogenesis can largely be explained based on MM-stroma interactions, investigating miRs in this context will be more attractive in terms of detection of potential therapeutic targets. Notably, the role of miRs in modulating tumor cells interaction with stroma for maintaining tumor load, shaping metastasis and helping development of tumor-associated fibroblasts has been well highlighted [23,24].

**Table 1:** Expression and relevant regulatory functions of some investigated miRs in MM cells in the context of BMME components

**miRNA**	**Expression pattern**	**Function**	**Ref.**
miR-15a/16	Down-regulated	Regulating MM cell proliferation through AKT3, MAPKs and NFκB pathways and by targeting cyclin D1, cyclin D2 and CDC25A	[10]
miR-15a/16	Down-regulated	induction of BMSC-mediated drug resistance in MM cells, possibly through IL-6	[28,35]
miR-21	Up-regulated	Induction of BMSC-mediated drug resistance in MM cells partially through NFκB pathway and by targeting RhoB	[27]
miR-199a-5p	Down-regulated	Regulating bone marrow angiogenesis through targeting HIF-1α in MM cells and modulating interaction with BMSCs and bone marrow endothelial cells	[32]
miR-135b	Up-regulated	Up-regulated in MM bone marrow mesenchymal stem cells following interaction with MM cells, impairing osteogenesis by targeting SMAD5	[34]
miR-199a, -24-3p, 15a-5p, 16-5p	Down-regulated	Down-regulated in MM mesenchymal cells, contributing to impaired osteogenesis	[33]

Lin J et al. established a model comprising lymphoma cells adhered to BMSCs (HS-5 cell line) or lymph node stroma (HK cell line) [25,26]. Using global gene expression arrays, they found a remarkably changed expression pattern of several miRs in lymphoma cells adhered to stroma cells. More noticeably, miR-548m was underexpressed in adhered lymphoma cells but its ectopic overexpression suppressed stroma-induced clonogenic growth and drug resistance, and triggered apoptosis. Although very limited research as such has been performed in MM, obtaining closely related findings should not be unexpected. Xudong et al. studied the expression pattern and function of miR-21 in MM cells adhered to BMSCs and found that miR-21 expression was increased in HMCLs adhered to BMSCs which was partly regulated through NFκB signaling pathway [27]. They also observed that bortezomib reduced miR-21 expression in MM cell-BMSC co-culture, however; it was not clear whether bortezomib targeted MM cells, BMSCs or both to down-regulate miR-21.

In a more recent study, Emanuela et al. also demonstrated that HMCLs with low miR-21 expression (INA-6) displayed a high expression of miR-21 when adhered to BMSCs. miR-21 inhibition significantly decreased viability and clonogenic growth of MM cells but in stroma-free conditions [18]. Of note, they showed that anti-tumor activity in MM cell-BMSC co-cultures was induced only when MM cells were transfected with miR-21 inhibitor but not the BMSCs. This suggests that miR-21 inhibition in MM cells counteract protective function of BMSCs in co-culture experiments.

Roccaro et al. reported a miR signature in MM cells indicating downregulation of miR-15a/16-1 [10]. Specifically, they showed that miR-15a/16-1 ectopic expression reduced DNA synthesis, cell cycle and proliferation in MM cells, and decreased their adhesion to BMSCs. However, this study did not show the expression pattern and function of miR-15a/16-1 in MM cells following adhesion to BMSCs.

Later on, Hao et al. also observed that BMSCs maintained survival of MM cells and protected them from bortezomib-induced apoptosis through suppressing miR-15a in MM cells [28]. Of note, miR-15a/16-1 is located on chromosome 13q14 (a region most commonly deleted in MM), and complete absence of miR-15a/16-1 has been found in MM cells with this deletion [10]. On the other hand, another study revealed a heterogeneous expression pattern not correlated with chromosome 13 status [29]. Interestingly, it is reported that in B-chronic lymphocytic leukemia (B-CLL), miR-15a/ 16–1 are hosted by *DLEU2*, a regulatory gene which is frequently deleted in CLL leading to repression of miR-15-a/16 (thus cell cycle progression, proliferation and anti-apoptosis) [30]. It remains to be determined whether the above gene also hosts miR-15/16-1 in MM. BMSCs in MM could express miRs which should also be considered in MM pathogenesis. Reportedly, expression of some miRs (miR-16, miR-223, miR-485-5p and miR-519d) and adhesion molecules genes in MM-BMSCs is higher than in normal counterparts [31]. Furthermore, some miRs have been reported to be modulated in BMSCs or MM bone marrow mesenchymal stem cells following interaction with MM cells contributing to angiogenesis induction (199a-5p) [32] or osteogenesis impairment (135b, 24-3b, 15a-5p) [33,34] (see also Table 1).

### Mediators/targets of miR-related signaling in MM in the context of BMME

**Figure 1:**
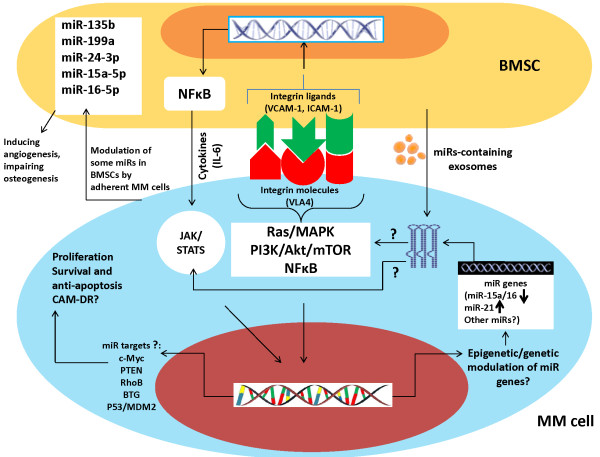
**Postulated schematic model indicating how BMSCs might influence expression and function of miRs in MM cells.** Following adhesion, integrin-mediated signaling in MM cells triggers activation of various pathways (mostly NFB, PI3K/Akt/mTOR, and Ras/MAPK). It is still not known whether miRs associate with these pathways, whether modulation of miR gene expression occurs through these pathways, and whether they induce some epigenetic mechanisms controlling expression of miRs. It has also been shown that BMSCs can transfer miR-containing (15a) exosomes into MM cells to induce cell growth and proliferation. IL-6 has also been demonstrated to mediate miR-15a suppression in MM cells following adhesion to BMSCs, but how this cytokine triggers such a response is not clear. Moreover, targets reported for some miRs (miR-21) in MM cells, were not explored in BMME context, hence the potential targets of miRs in MM cells adhered to BMSCs are not well characterized yet nor is there any information showing how these targets are affected by a putative integrin-miR axis. To identify potential targets of miRs in MM cell-BMSC interaction, further exploration is required. Additionally, adhesion of MM cells to BMSCs has been shown to modulate some miRs in BMSCs, leading to other disease-related complications such as angiogenesis and defective osteogenesis. However, to identify potential targets of miRs in MM cell-BMSC interaction, further exploration is required.

To understand the biology of miRs in MM cells in BMME, expression and function of miRs should be explored in above context, because only in that case can we trace any miRs link with bone marrow milieu-induced events especially CAM-DR. In fact since the effects of MM cells-BMSCs interaction are mutual, in experimental and animal models of MM-BMSC interaction it should be characterized what signals are triggering miRs expression alterations in MM cells (illustration at Figure 1). Does the BMSCs induce miR expression changes in MM cells through induction of adhesion-related signals in latter cells?, does it happen due to influence of some cytokines/mediators released by BMSCs?, is the altered expression of miRs in MM cell-BMSC co-cultures a function of co-operative interaction (miR change in both cell types) or the inductive effect of one cell type upon the other?, and finally, are miRs associated with integrin signaling pathways in MM cells following adhesion to ECM proteins such as FN?. To find answers, several studies in recent years have provided some clues. For instance, it has been reported that IL-6 secreted by BMSCs increases drug resistance and reduces apoptosis of MM cells through suppression of miR-15a/16-1 [35], yet how IL-6 performs and what the miR targets are, have not been mechanistically characterized. More interestingly, another study showed that BMSCs transfer exosomes containing miR-15a into MM cells inducing their proliferation and survival [36]. Indeed miR-containing exosomes have been reported to play important roles in pathogenesis of various cancers [37]. RhoB, BTG, and PTEN have been indicated to be targets of miR-21 in MM cells [18], however; these findings were obtained in a stroma-free condition, thus not highlighting their real function in the context of bone marrow milieu. Lwin T et al. using *in vitro* and *in vivo* investigations found that stromal cells contributed to sustained c-Myc upregulation and miR-548m downregulation through a c-Myc/miR-548m feed-forward amplification loop leading to lymphoma cell growth and proliferation [26]. They also showed that miR-548m by directly targeting HDAC6, linked HDAC6 upregulation with lymphoma cell survival and drug resistance. Whether c-Myc can be similarly involved in miR-mediated functions in BMSC-MM cell interaction needs to be further investigated. The more important point in above study is the involvement of an epigenetic mechanism in controlling miR-induced responses. Indeed, epigenetic mechanisms have also been suggested to control miR-associated functional responses in MM cells [16]. Intriguingly, miRs 192, 194 and 215 (transcriptional targets of p53) were found to be hypermethylated in MM cells explaining their lower expression in MM than in MGUS [8,17]. However, it would be more interesting to investigate if BMSC or ECM triggers such regulatory mechanisms in MM cells, as given so, we may understand whether altered expression and function of some miRs following MM cell-BMSC/ECM interaction could underlie such events as CAM-DR. With our current knowledge, we still don’t know how miRs are modulated in MM cell-BMME context, and which critical oncogenes or tumor suppressor genes are targeted by miRs in this context.

### Concluding remarks and future prospects

Research on biology and function of miRs tends to achieve a hot spot in the field of MM therapy, with some evidence to introduce miRs as promising therapeutic targets in MM [13]. However, most findings are indicative of MM cells drug responses in the absence of BMME irrespective of the fact that BMME plays a prominent role in the pathogenesis of MM. To this end, several *in vitro* and *in vivo* investigations have yielded important clues that some miRs (miR-21 and miR-15a/16-1) might play role in stroma-mediated drug resistance of MM cells but the specific targets and the underlying mechanisms are not clear yet. Moreover, the possibility of involvement of other miRs, their potential targets, and involved signaling pathways, warrants more in-depth research. As a matter of fact, reinforcing our knowledge of miRs expression and function in MM cell-BMME interaction will help us to find *potential drug targets* to overcome CAM-DR, which has been suggested to build the *intrinsic* (*de novo*) drug resistance and contribute to *acquired* drug resistance over time in MM patients [38-41].

## Abbreviations

Akt: Also known as protein kinase B is a serine/threonine-specific protein kinase; BMSC: Bone marrow stromal cell; BTG: B cell translocation gene; CAM-DR: Cell adhesion-mediated drug resistance; ICAM-1: Intercellular cell adhesion molecule-1; JAK/STAT: Janus kinase/Signal transducer and activator of transcription; MAPK: mitogen activated protein kinase; MDM2: Minute double minute 2; mTOR: Mammalian target of Rapamycin; NFκB: Nuclear factor kappa-light-chain-enhancer of activated B cells; PI3K: Phosphatidylinositol-4,5-bisphosphate 3-kinase; PTEN: Phosphatase and tensin homolog; Ras: A member of small GTPase family; RhoB: Ras homolog gene family member B; VCAM-1: Vascular cell adhesion molecule-1; VLA4: Very late antigen 4.

## Competing interest

The authors declare that they have no competing interest.

## Authors’ contribution

JA participated in the design and drafted the review, LQ provided critical revision, HC designed, supervised the study, participated in drafting the review and provided critical revision; All authors read and approved the final manuscript.
